# Investigating the Effects of Personality on the Safety Behavior of Gold Mine Workers: A Moderated Mediation Approach

**DOI:** 10.3390/ijerph192316054

**Published:** 2022-11-30

**Authors:** Li Yang, Sumaiya Bashiru Danwana, Fadilul-lah Yassaanah Issahaku, Sundas Matloob, Junqi Zhu

**Affiliations:** 1 School of Economics and Management, Anhui University of Science and Technology, Huainan 232001, China; 2 School of Mathematics and Big Data, Anhui University of Science and Technology, Huainan 232001, China

**Keywords:** big five personality traits, safety behavior, safety competency, management safety commitment, mining, moderated mediation, Ghana

## Abstract

Based on prior research on the relationship between personality and safety behavior, we construct a moderated mediation model that tests the effects of each of the Big Five personality traits (openness, conscientiousness, agreeableness, extraversion, and neuroticism) on the safety behavior of gold miners in Ghana. The model included safety competency as a mediator between the five personality traits and safety behavior. Management commitment to safety was used as a moderator to test the strength of the mediation of safety competency. Data was collected from 344 individuals employed across six large-scale gold mining companies in Ghana using a questionnaire survey. Amos 26 was used to conduct confirmatory factor analysis. The hypotheses were tested using Hayes PROCESS macros models 4 and 7 on SPSS 26. Findings show that openness and extraversion have an insignificant direct influence on safety behavior. Neuroticism negatively affects safety behavior. In contrast, conscientiousness and agreeableness positively affect safety behavior. Mediation analysis revealed that safety competency partially mediates the relationships between (1) conscientiousness and safety behavior and (2) agreeableness and safety behavior. The relationship between extraversion and safety behavior is fully mediated by safety competency. Additionally, we discovered that management safety commitment has a significant conditional indirect effect (Index of moderated mediation = 0.168 95% CI = [0.122;0.222]) on the relationship between conscientiousness and safety behavior through safety competency. Management safety commitment also significantly moderated (Index of moderated mediation = 0.075 95% CI = [0.021;0.120]) the relationship between agreeableness and safety behavior through safety competency.

## 1. Introduction

For a long time, worker health and safety of the working population has been a worldwide priority. The quality of a person’s working life cannot be measured without considering the importance of a safe work environment. Sustainable Development Goals (SDG) 3 and 8 of the United Nations urge companies to prove their worth by improving employees’ access to decent employment and advocating for their safety and well-being. Workers in mines are subject to a wide range of potential dangers and hazards due to the nature and complexity of their job [1]. Despite significant efforts in many countries to lower the rates of death, injury, and disease among worldwide mineworkers, mining remains the most hazardous occupation [2,3,4] when the number of individuals susceptible to accidents and injuries is considered [5,6]. According to The World Counts [7], about 15,000 miners lose their lives yearly. The situation is believed to be the worst in developing countries [8], with low adherence to health and safety rules. The harmful impacts of mining on miners’ well-being cannot be ignored, despite its significant economic advantage [1]. Mine workers frequently encounter hazardous situations and a lack of safety safeguards, which leads to accidents [9]. These accidents have repercussions for the mines and take a psychological toll on the workers [10].

In Ghana, mining is among the top economic contributors to investment, foreign trade, and employment. Despite its many benefits to society, the sector is widely known for increasing cases of occupational injuries and accidents. According to Stemn [6], the number of injuries in Ghana’s mining sector is significantly higher than in other major mining nations like Australia and the United States of America. Despite Ghana’s position as the biggest producer of gold in Africa and seventh biggest in the world, very little is known about the industry’s safety record. Stemn [6] estimates that the Ghanaian mining sector records five fatalities and 51 serious injuries yearly. The Minerals Commission of Ghana [11] reported an average of 333 mining-related accidents and 1915 minor injuries between 2008 and 2014. Opoku, Kosi, and Degraft-Arthur [12] reported 155 injuries, 19 serious accidents, and four fatalities in Ghana’s mining sector in 2015.

Accidents harm a company’s image and profitability since they result in medical expenses, time away from work, legal fees, compensation claims, destroyed machinery, and other losses [13]. Preventing accidents and incidents is a fundamental premise of occupational health and safety. It is imperative to identify any potential contributing variables before an actual accident occurs [14,15]. Finding the primary causes is the first step in preventing these accidents. Some of these factors have been looked at in earlier research. Among the factors influencing the likelihood of injury are human errors [4,16] and unsafe behavior [17]. Accidents involving work-related injuries and casualties are almost always the consequence of human errors [18]. According to Heinrich, unsafe behavior is to blame for 88 percent of injuries [19,20]. Promoting safe behavior is a sure way to prevent workplace accidents [21]. The science of behavior-based safety was developed to find solutions to the problem of unsafe behavior and ways in which human behavior can be changed to prevent accidents [5,22].

Safety behavior has long piqued the attention of researchers in the domains of psychology and organizational behavior. It has emerged as a key tenet of current health and safety management theory [23]. Safety behavior includes all individual actions connected to the organization’s safety. When an individual or group of workers takes steps to reduce the likelihood of fatalities and injuries, they engage in “safety behavior”, as defined by [24]. Safe work behaviors are measurable because they are intrinsically linked to psychological aspects. Neal et al. [25] assert that safety behavior is reflected in safety participation and safety compliance. Safety participation involves voluntary actions that advance safety inside the company as opposed to safety compliance, which includes mandatory safety behaviors [26]. Safety compliance and safety participation are jointly referred to as safety behavior in the present research.

In the mining industry, safety compliance includes, but is not limited to, frequently inspecting safety equipment and using the proper personal protective equipment while at work and wearing trackers and sensory devices when working underground. This behavior is considered mandatory in the safety standards and procedures of mining operations and must be carefully executed by workers. Behaviors like suggesting effective ways to handle safety based on one’s expertise in safety briefings, encouraging coworkers with low safety awareness to use appropriate safety equipment at work, and asking coworkers to actively take part in safety training are all safety participation behaviors that are not covered by safety management regulations but are meant to increase awareness of safety. Additionally, it is the workers’ effort motivated by their understanding of the value of safety. Notably, with underground mining activities, employees and employers are expected to engage in safety compliance and safety participation. Employees are obliged to execute proper safety at work daily, while employers oversee the safe operation and the company’s overall safety framework.

Clarke and Robertson [27] and Beus, Dhanani, and McCord [28] conducted a meta-analysis that suggested and empirically validated that personality traits have an impact on occupational accidents and that these traits are expressed through workers’ safety behaviors. They pointed out that people’s safety behaviors at work are fundamentally determined by their personalities and are thus directly linked to the occurrence of accidents. Some employees are more likely to have an accident at work than others [16]. That is to say, a person’s personality qualities may make them more likely to act recklessly, which eventually results in accidents. Due to their low regard for safety, these people may engage in unsafe behavior. Research suggests that excluding those likely to cause accidents during the hiring process might decrease workplace accidents. Personality evaluation can be beneficial in detecting people likely to engage in unsafe behavior, but it is first necessary to understand how personality affects safety [29].

A fundamental understanding of the factors contributing to unsafe behavior and accidents is necessary to effectively implement safety measures in gold mines in Ghana. This research uses the Big Five personality traits as the main independent variables influencing safety behavior. The study develops a moderated mediation model to examine how different personality traits (openness, conscientiousness, extraversion, agreeableness, and neuroticism) influence safety behavior and if the connection is affected or changed when management commitment to safety and the safety competency of the individual is considered. There are several compelling reasons to delve into how the Big Five personality traits relate to safe behaviors. First, when trying to figure out why someone does what they do in response to information, it helps to look at their personality [30]. Second, the Big Five personality traits offer a practical theoretical structure for investigating how one’s unique traits affect the likelihood of engaging in unsafe behavior at work. The model of this study demonstrates how personality affects employees’ safety behavior through safety competency. Safety competency is a mediator between personality traits and safety behavior, while management safety commitment moderates the intervention of safety competency.

A significant contribution of this study is the mediating effect of safety competency and the moderating function of management safety commitment. Although safety competency, management safety commitment, and personality traits directly impact safety behavior, it is intriguing to look at how these factors interact. Fourth, this work makes an important addition to the literature on behavior-based safety by providing evidence for a moderated-mediation hypothesis, which has not been thoroughly studied in Ghana’s gold mining sector. Workers’ safety behavior is seldom studied by considering the factors we explored in this research, especially in a developing nation. Though research has been done in the Ghanaian setting, discussions of the intricate web of interactions that make up this study are rare. As a result, the study adds something unique to the increasing corpus of research on behavior-based safety.

The rest of the paper is structured as follows. The next section presents the theoretical background and hypothesis of the study. The methods section, Section 3, discusses the survey method, participants, measures, and data analysis mode used. Section 4 follows with an analysis and presentation of the results. Section 5 presents a general discussion of the results. The limitations of the study and directions for further research are presented in Section 6. The final section, Section 7, includes the conclusions drawn and the study’s policy implications.

## 2. Theoretical Background and Hypothesis

### 2.1. Personality and Safety Behavior

Personality is an intrinsic distinguishing feature that influences the behavior of a person [31]. It involves specific characteristics created through time and sets one individual apart from another [32]. Personality traits can influence high-risk activities and how people perceive danger and health [33]. Psychological personality trait theory holds that personality traits impact human behavior and are seen to be more or less consistent in scenarios and across a lifespan. The Big Five personality traits, also called the five-factor model [34], have emerged as the standard reference in the field of personality research [35]. The Big Five personality traits are neuroticism, extraversion, openness to experience, agreeableness, and conscientiousness. In this paper, we use these traits to categorize the personalities of miners.

Neuroticism describes a person’s emotional stability and how emotions, particularly unpleasant ones, are felt [36]. Nervous persons are more prone to hostile attitudes, despair, irritability, and emotional instability [34,37]. Extraversion evaluates social behavior and includes traits like assertiveness, adventurousness, and optimism [36]. People’s eagerness to engage in new experiences and ideas is captured by their openness to them. Therefore, people with the trait of openness tend to be imaginative, versatile, and dynamic [34]. Being agreeable includes qualities like selflessness, reliability, and friendliness [34]. Conscientiousness includes organizing and carrying out activities deliberately and methodically [34].

Several academics have tried to ascertain the connection between personality traits, safety behaviors, and work injuries in various industrial contexts over the last several decades. Refs. [14,28,38] found that traits of conscientiousness and agreeableness have a positive relationship with safety behavior. Conscientiousness is generally related to adherence to rules. In contrast, Wallace and Vodanovich [39] reported a negative relationship between conscientiousness and safety at work. Researchers found that people who score high on openness would be more inclined to take risks because they are more interested in discovering new ways and are less careful [29,40]. They are also more inclined to deviate from societal expectations, which may involve taking precautions like using the appropriate personal protective equipment [41]. Beus et al. [28] assert that there is little or no relationship between openness and work safety. Day, Brasher, and Bridger [42] discovered that people under stress have a tendency toward memory lapses, which makes them more likely to have an unintended accident at work. Neurotic personality traits, in general, constitute a significant threat to safety behavior in mines [2,22]. Hansen [13] and Chung [13,43] agree that extraversion and neuroticism correlate with accident occurrence. Wallace and Chen [44] add that extraversion has a negative relationship with safety.

Although there is a large body of literature on the connection between personality traits and safety behavior, some issues must be resolved before the study findings may be used for accident prevention in a particular cultural setting. It is indisputable that an individual’s personality has a role in their actions, even if it is challenging to draw a firm conclusion that the different personality traits consistently correlate with safety behavior. Jong-Hyun et al. [45] have thus proposed that ongoing studies on the correlation between personality traits and safety behaviors will result in significant breakthroughs in our knowledge of these subjects. We generate the following hypothesis based on the arguments above:

Hypothesis 1 (H1):

**H1a:***Openness is negatively related to safety behavior.*
**H1b:***Conscientiousness is positively related to safety behavior.*
**H1c:** *Extraversion is positively related to safety behavior.*
**H1d:** *Agreeableness is positively related to safety behavior.*
**H1e:** *Neuroticism is negatively related to safety behavior*.

### 2.2. Safety Competency as a Mediator

A mediator is a variable that influences the way an independent variable affects a dependent variable [46]. It reveals how or why two variables are related. It is possible that the relationship between independent and dependent variables no longer exists once the impact of a mediator is taken into consideration. Mediation analysis is used to test the effect of the mediator on the relationship between the independent and dependent variables. In the context of our study, we test the mediation effects of safety competency on the relationship between each of the Big Five personality traits and safety behavior.

The term “competency” refers to a set of predetermined actions that serve as a systematic framework for recognizing, creating, and measuring specific behaviors that help people perform their jobs effectively. Competent people possess knowledge in their professions, an understanding of any theoretical approaches, practical experience using those ideas in various circumstances, the ability to solve problems, and the ability to express concerns to others clearly and understandably. Additionally, competencies may affect a person’s behavior, and lesser skills might result in errors and unfavorable results [47].

The capacity to practice the mix of education, skills, experience, and information to carry out a task safely is known as safety competency [48,49]. It is an innate characteristic of an individual that correlates with a greater degree of safety [50]. These safety competencies are proficiency in safe behavior strategies and abilities built on a solid foundation of safety knowledge [47]. They are essential concerning the necessary degree of safety and achieving adequate job efficiency. The first step in developing a comprehensive safety program is identifying the core competencies necessary for carrying out specific duties and tasks. To be successful in the safety-related area, individuals need to cultivate a particular set of skill sets pertinent to their job responsibilities and company goals. In high-risk industries, the absence of safety competency might have catastrophic effects. A lack of fundamental safety competency might result in multiple injuries [50]. Good and applicable safety skills and competencies should shape high-quality safety behaviors.

An individual’s personality may influence both their safety competency and safety behavior. Personality affects how prepared and conscious of safety each employee is. This is because a person’s personality impacts their degree of ambition, which defines the strength of desire for accomplishments. It lays out a plan for undertaking tasks in relation to one’s level of engagement and attentiveness. It also determines a person’s demand for stimulation, levels of distress, and style of information processing, which determines one’s unique way of viewing the world and influences one’s assessment of risks and therefore the magnitude of the risk taken.

**Figure 1:**
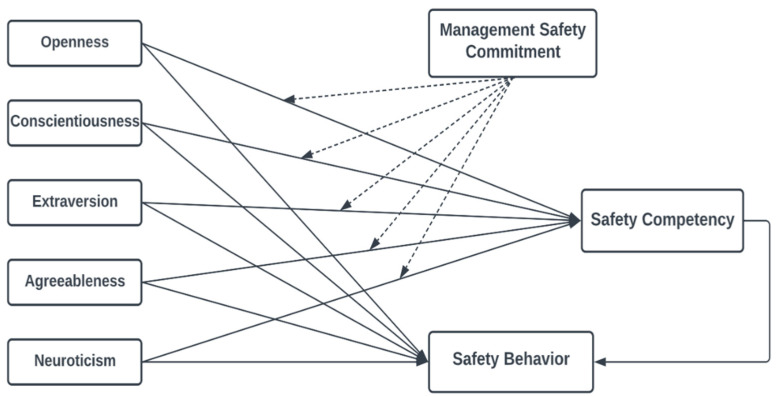
Conceptual Framework.

There is an apparent link between personality, safety competency, and safety behavior which has been explored in research. To cover this gap, we use safety competency as a mediator in the relationship between safety behavior and personality traits (Figure 1). We hypothesize that:

Hypothesis 2 (H2):

**H2a:***Safety competency mediates the relationship between openness and safety behavior.*
**H2b:***Safety competency mediates the relationship between conscientiousness and safety behavior.*
**H2c:** *Safety competency mediates the relationship between extraversion and safety behavior.*
**H2d:***Safety competency mediates the relationship between agreeableness and safety behavior.*
**H2e:** *Safety competency mediates the relationship between neuroticism and safety.*

### 2.3. The Moderating Role of Management Safety Commitment

A moderator is a third variable that affects how strongly the independent and dependent variables relate [51]. The direction or existence of a link between variables is affected by a moderator. As the third variable affects the relationship between the two others, the influence of the moderator is also known as an interaction. We introduce management safety commitment as a moderator to find out if the safety commitment of management strengthens or weakens the effect of personality on safety competency.

Management is accountable for taking all necessary precautions to provide a safe working environment and address potential risks [10,52]. The effectiveness of a company’s safety policies is significantly influenced by management’s commitment to safety [5]. When safety regulations are disregarded or regularly broken, the importance of safety is misrepresented. There are also times when a company has to decide between safety and its production objectives. The capacity to do work safely requires the availability of resources, which management should provide while establishing goals for all aspects of operation, safety included [10]. Management is a key component of safety behavior interventions [52].

Workers are usually asked about their satisfaction, expectations, attitudes, and behaviors to evaluate management safety commitment performance [53]. It is evident that how employees view managerial commitment is crucial. Within the idea of a safe workplace, the term is often described as the length to which management is thought to prioritize, act on, and articulate safety concerns [54]. Management’s commitment to safety must materialize concretely; it cannot just be lip service. Fruhen et al. [53] assume that workers will evaluate management commitment based on the extent to which they see the manager putting out effort in their capacity as a leader. Managers who emphasize the value of safety in their communications with staff and who devote funds to its enhancement will be seen as having their staff’s best interests at heart. According to O’Toole [55], workers’ safety attitudes may be influenced by management’s ability to convey good standards and expectations, which may incite them to prioritize safety. The priority of management should be to educate and provide a safe working environment. They also stress the need for clear safety regulations and rewarding employees for safe work habits [10].

In potentially dangerous workplaces like mining sites, it is standard to create safety manuals focusing on the procedures and regulatory obligations outlined by the relevant law enforcement agencies. The company must choose if it must be put into effect or whether it may merely be written about by acting as role models for employees, acknowledging best practices, and urging them to follow stringent safety regulations. They may revitalize a company’s safety management strategy and encourage workers to take more protective measures for themselves. Erickson [56] agreed, noting the importance of management’s hands-on, sincere, and constant commitment to creating a risk-free, healthy workplace for their workers.

We have mentioned that the personality traits of a person have an impact on their safety competency. We believe that the relationship between personality traits and safety competency can be moderated by management commitment to safety. This is because the level of safety commitment of a company’s management can affect the competence of workers with different personalities in different ways. For example, workers with the trait of openness are likely to break safety regulations even if there have a high level of safety competency. However, with a management that is highly committed to ensuring the safety of workers, they may conform to safety measures. To investigate the moderating role of management safety commitment, we hypothesize that:

Hypothesis 3 (H3)

**H3a:** *Management safety commitment moderates the relationship between openness and safety competency*
**H3b:** *Management safety commitment moderates the relationship between conscientiousness and safety competency.*
**H3c:** *Management safety commitment moderates the relationship between extraversion and safety competency.*
**H3d:** *Management safety commitment moderates the relationship between agreeableness and safety competency.*
**H3e:** *Management safety commitment moderates the relationship between neuroticism and safety competency.*

We also propose a moderated mediation model of these linkages based on the discussion above. Moderated mediation, also known as conditional indirect effect, tests whether the indirect effect (mediator) is conditional on the values of a moderating variable [57]. We contend that management commitment to safety moderates the effect of personality on safety behaviors via safety competency (Figure 1). A high or low level of management safety commitment is expected to have an impact on the indirect association between personality features and safety behavior through safety competency. To ascertain the direction of this connection, we hypothesize that:

Hypothesis 4 (H4)

**H4a:** *Management safety commitment moderates the strength of the mediated relationship between openness and safety behavior via safety competency.*
**H4b:** *Management safety commitment moderates the strength of the mediated relationship between conscientiousness and safety behavior via safety competency.*
**H4c:** *Management safety commitment moderates the strength of the mediated relationship between extraversion and safety behavior via safety competency.*
**H4d:** *Management safety commitment moderates the strength of the mediated relationship between agreeableness and safety behavior via safety competency.*
**H4e:** *Management safety commitment moderates the strength of the mediated relationship between neuroticism and safety behavior via safety competency.*

## 3. Methods

### 3.1. Survey and Participants

Data was gathered through a questionnaire survey. The questionnaire consisted of 50 items divided into two parts. The first part included five questions about the demographics of the respondents, including gender, age, relationship status, level of education, and working experience. The second part of the questionnaire was used to collect data on the personality traits of respondents, safety behavior, safety competency, and their opinions of management commitment to safety. Participants were asked to rate their agreement with each statement on a 5-point Likert scale (1—strongly disagree to 5—strongly agree).

A convenience sampling approach was used to select research participants. All survey respondents were employees of six large-scale gold mining companies in the Western North, Western, and Ashanti regions of Ghana. Participants included workers below the supervisory level. The study’s participants were made aware that their participation was entirely voluntary and that they might revoke their consent at any moment without giving a reason. The survey was made available in both hardcopy and online formats. The choice of a hardcopy or online survey was left to the respondent to choose based on which was more convenient for them. Six people were recruited as research assistants to help collect data by being ready to answer any questions from survey participants and retrieving completed questionnaires.

The study and survey received approval from the research ethics committees of Anhui University of Science and Technology. Before data collection began, consent and data confidentiality agreements were signed by the mining companies participating in the survey. The survey was carried out at two time intervals. The first round of data collection, which lasted two months (April to May 2022), collected data on respondents’ demographics, personality traits, and safety behavior. The second round collected data on the moderator (management safety commitment) and the mediator (safety competency). Twenty questionnaires were used in a pilot test, which confirmed that the questions were simple, legible, and easy to complete.

Out of the 500 questionnaires distributed, the survey received responses from 357 mining employees, yielding a 71.4% response rate over four months (April to July 2022). Data from 13 of the 357 replies were judged unreliable and excluded from the following data analysis. Ultimately, 344 sets of responses were determined to be reliable. Using a paired sample *t*-test of both surveys, we found that Participants who did not participate in the second round of data collection did not vary significantly from those who participated in the analysis of demographic factors, suggesting that the sample loss in this study was truly random.

Most respondents were male (76.5%), and the age demographic showed that 43.6% of employers are in the age group of 30–40 years and above 60% are married. Level of education indicated a majority of bachelor’s degrees, followed by nearly 30% of respondents having master’s degrees. Over 70% of the respondents have worked in the company for 2 to 10 years. In Table 1, demographic details of valid responses are presented.

### 3.2. Measures

The scale items used in this analysis were derived from previously published research. The language of several of the items was changed to reflect the Ghanaian gold mining industry’s unique setting and provide face and content validity. The language of all the items was English. Below is a detailed description of the measure used for each variable.

1. Personality: A 20-item version of the personality trait inventory adopted from [58,59] was used to collect data on personality variables. Five questions each were used to assess openness (Cronbach alpha = 0.779), conscientiousness (Cronbach alpha = 0.752), agreeableness (Cronbach alpha = 0.789), extraversion (Cronbach alpha = 0.815), and neuroticism (Cronbach alpha = 0.872). A 5-point Likert scale ranging from 1 (strongly disagree) to 5 (strongly agree) was provided to measure how well each item related to respondents. Respondents were asked to complete the statement “I see myself as a person who is” with the following samples of personality traits; Enthusiastic, Outgoing, Helpful, Sympathetic, Efficient, Organized, Nervous, Moody, Creative, and Broadminded.
2. Safety Behavior: As mentioned earlier, safety participation and safety compliance are jointly referred to as safety behavior in this study. Numerous measures have been established to evaluate safety participation and compliance, with the scale proposed by [25,26] being the most used. This scale has been updated for usage in light of the respondents to the survey. Nine items were used to evaluate safety behavior. Out of the nine, five items measured safety participation, while four measured safety compliance [25]. Participants scored how much they agreed or disagreed with each item on a five-point scale varying from 1 (Strongly Disagree) to 5 (Strongly Agree). The Cronbach alpha (α) of all the nine items on safety behavior is 0. 869. An example of the items are “I am always mindful of safety at work”, “I wear the right personal protective equipment before I start my work”, and “I raise safety concerns when necessary”.
3. Safety Competency: The safety competency of the individual was recorded with eight items using a 5-point Likert scale. The scale reliability test revealed a high Cronbach alpha value of 0.95. Items were adopted and developed from Bensonch et al. [60]. Some of the items in this variable include “I possess the expertise required for the efficient completion of duties”, “I am knowledgeable about the rules, practices, and directions about occupational safety”, and “I am aware of the operational guidelines and safety needs specific to the position I occupy”.
4. Management Safety Commitment: Respondents’ perspective of management’s ability to enable and empower employees to act more safely was assessed using eight items according to participants’ perspectives. Questions on management safety commitment were adapted from [23] and measured on a five-point Likert scale (1 (strongly disagree) to 5 (strongly agree)). In the current analysis, the scale’s alpha coefficient is 0.84. The statements “Mangers take appropriate measures when notified of unsafe practices” and “My impression is that executives are prepared to forgo safety to increase output” are samples of the items.

### 3.3. Control Varaibles

The study included all the demographic variables (gender, age, relationship status, education, and work experience) as control variables. Gender was coded as “1 = Male and 2 = Female”. Age was coded as “1 = 18–29, 2 = 30–40, 3 = 41–50, and 4 = 51–60”. Relationship Status coded as “1 = Single, 2 = Married, 3 = Separated, 4 = Divorced, and 5 = Widowed”. Education as “1 = Senior high or below, 2 = Bachelors, 3 =Masters, 4 = Doctoral or higher”. Finally, Work Experience was coded as “1 = Less than two years, 2 = 2 to 5 years, 3 = 6 to 10 years, and 4 = More than 10 years”.

### 3.4. Data Analysis

Data were analyzed using SPSS 26 and Amos 26. SPSS 26 was used to clean and filter the data and conduct an exploratory factor analysis, correlation, and descriptive analysis. Missing values, outliers, and normality analysis of the data were all included in the cleaning and screening of the data. Variables have a weakly skewed distribution, according to the test for normality. No multivariate outliers were found. Exploratory factor analysis (EFA) led to the deletion of 12 items due to low factor loadings (loadings below 0.5). Kaiser-Meyer-Olkin (KMO) and Bartlett’s Test of Sphericity were also conducted to check if the factors used in the study were suitable for factor analysis. KMO value must be above 0.5 and closer to 1 for significance, while the significance of Bartlett’s Test of Sphericity should be below 0.05. The result of the KMO test was 0.806, and the significance of Bartlett’s test was 0 (Approx. Chi Square 1080.572, df 15), which shows that it is plausible to conduct factor analysis.

AMOS 26 was used to conduct a Confirmatory factor analysis to assess the model’s fitness to the data. The model was validated by performing tests on the measurement model and comparing the values to the recommended model fit indexes proposed by [61]. Several models were tested, and the best fit was selected.

Rather than the conventional mediation analysis strategy of [51] or the popular structural equation modeling (SEM) approach, we estimate the mediating and the moderated mediating effects estimated using model 4 and model 7 of Hayes PROCESS macro for SPSS [62,63]. The process involved using a bias-corrected bootstrapping using 5000 bootstrap samples at a 95% confidence interval. The bootstrap test is considered significant at 0.05 if there is no zero within the upper and lower confidence intervals (CI). We chose the PROCESS macro over SEM because it is a more recent method for data analysis that provides a graphical depiction of moderated correlations. Additionally, this approach generates a moderated mediation index that indicates whether the study model should be accepted or rejected.

## 4. Results

### 4.1. Test for Common Method Bias

There is an increased risk of common method bias when researchers use the same setting or items with comparable characteristics [64]. We gathered data from two periods to account for any possible Common Method Variance (CMV). Every poll had a two-month gap between them. To protect the privacy of our participants, we did not provide any identifying information about them. To lessen the possible influence of inattentiveness, the researcher included a mix of negatively and positively worded questions on the questionnaire. This lowers the possibility of participants giving incorrect or misleading responses.

We also conducted Harman’s one-factor test using SPSS. Common method bias is present if the percentage of variation explained by a single component in the analysis is more than 50 percent [65]. The total variation explained by a single component is 37.8%, which is well below the 50% cutoff for avoiding common method bias. Since no primary component appeared or accounted for more than half of the variation, the result of Harman’s one-factor test suggests that common method bias is not a concern in this investigation. This evidence points to the absence of common method bias in the data [23].

### 4.2. Correlation and Descriptive Statistics

Descriptive statistics showing the standard deviations (SD), means(M), and inter-variable correlations (r) were computed on SPSS26 and presented in Table 2 below. Table 2 informs that there are significant positive correlations between safety behavior (SD = 0.633, M = 4.353) and conscientiousness (SD = 0.721, M = 4.13, r = 0.642, *p* < 0.01), extraversion (SD = 0.951, M = 3.442, r = 0.172, *p* < 0.01), agreeableness (SD = 1.009, M = 3.070, r = 0.483, *p* < 0.01), and neuroticism (SD = 0.665, M = 3.463, r = 0.805, *p* < 0.01). Of the five personality traits, only openness (SD = 1.088, M = 2.825) had an insignificant correlation with safety behavior, signifying no relation between the two. We also notice that the correlation between safety behavior and safety competency (r = 0.580) is positive and significant at 0.01. Correlations between safety behavior and management safety competency (r = 0.722, *p* < 0.01) show how closely and significantly these variables relate. Lastly, we also observe that safety competency positively relates to management commitment to safety (r = 0.539, *p* < 0.01).

### 4.3. Confirmatory Factor Analysis

We constructed and tested three models to find the suitable model fit for our data. First, we tested an eight-factor model, which included five variables to measure personality traits, safety behavior, safety competency, and management safety commitment. The second was a five-factor model where personality traits were converted into one variable, and safety behavior was split into two (safety participation and safety competency). The third, a four-factor model, included personality traits as a single variable, safety behavior, safety competency, and management commitment to safety. Model fit was evaluated using normed Chi-square (χ^2^/df), comparative fit index (CFI), the goodness of fit index (GFI), Tucker-Lewis index (TLI), root mean square error of approximation (RMSEA), and root mean squared residual (SRMR). Table 3 shows the acceptable limits for each model fit indices used.

As evidenced by values in Table 4, the eight-factor model is the right fit for the data with χ^2^/df of 1.395, CFI of 0.963, G.F.I. of 0.917, TLI of 0.956, RMSEA of 0.032, and SRMR 0.015. Model fit indices are all within acceptable limits shown in Table 3, meaning the model we used for the study fits the data perfectly.

### 4.4. Hypothesis Testing

We tested hypotheses 1 and 2 using the PROCESS macro model 4 and a bias-corrected bootstrapping using 5000 bootstrap samples. Table 5 displays the results. The first thing we did was examine the impact of the control variables (gender, age, relationship status, education, and work experience), and we found that none of them had any real significance. Therefore, we did not consider the control variables in the PROCESS analysis. Table 5 presents the standardized estimates of the total direct and indirect effects (mediation) between the variables.

The analysis tests the mediation effects of safety competency on the relationship between the Big Five personality traits (openness, conscientiousness, extraversion, agreeableness, neuroticism) and safety behavior. The results revealed an insignificant direct effect (β = −0.001, *p* > 0.05) of openness on safety behavior. Even though the effect is negative, as hypothesized in H1a, the result lacks statistical significance. Therefore, H1a is not supported. Furthermore, the indirect effect (β = 0.017, *p* > 0.05, CI = [−0.017;0.086]) of the relationship between openness and safety behavior is found to be positive but insignificant. With safety competency as a mediator, the relationship between openness and safety behavior is insignificant, giving us justification that H2a is also not supported.

Conscientiousness has a significant positive effect on safety behavior in the presence of safety competency (direct effect: β = 0.564, *p* < 0.001), supporting H1b. There is also a significant positive effect of conscientiousness on safety behavior through safety competency (indirect effect: β = 0.116, CI = [0.070;0.182]). The indirect effect is significant because zero is not included in the confidence interval. Given the significant direct and indirect relations between conscientiousness and safety behavior, safety competency partially mediates the relationship between conscientiousness and safety behavior, giving support to H2b.

Extraversion has a positive insignificant direct effect (β = 0.115, *p* > 0.05) and a positive significant indirect effect (β = 0.168, *p* < 0.05, CI = [0.132;0.205]) on safety behavior. This result does not support H1c, which proposed a positive relationship between extraversion and safety behavior. H2c is supported because there is a significant indirect effect (zero is not within the confidence interval), signifying that the relationship between extraversion and safety behavior is mediated by safety competency. Since the direct effect is insignificant and the indirect effect is significant, there is full mediation.

Hypothesis 1d tests the relationship between agreeableness and safety behavior. The results in Table 5 show that the direct effect (β = 0.108, *p* < 0.01) of agreeableness on safety behavior is positive and significant, supporting H1d. The mediation effect (β = 0.195, *p* < 0.01, CI = [0.130;0.268]) of safety competency on agreeableness and safety behavior is significant. The relationship between agreeableness and safety behavior is partially mediated by safety competency, with a significant direct and indirect effect supporting H2d.

Neuroticism negatively impacts safety behavior in safety competency (β = −0.271, *p* < 0.01), supporting H1e. With the influence of safety competency, there is no relationship between neuroticism and safety behavior (β = −0.002, *p* > 0.05, CI = [−0.002;0.006]). The indirect effect is insignificant because there is a zero within the confidence intervals. Therefore, safety competency does not mediate the relationship between neuroticism and safety behavior; H2e is not supported.

To test Hypothesis 3, we proceeded to test for the moderating effect of management safety commitment on the relationship between personality traits and safety behavior. We used the Hayes PROCESS macro model 7 for the moderation analysis.

Hypothesis 3a proposes that management safety commitment moderates the mediation between openness and safety competency. Results show that the interaction term is insignificant; management safety commitment does not moderate the relationship between openness and safety competency. Therefore, H3a is not supported.

**Figure 2:**
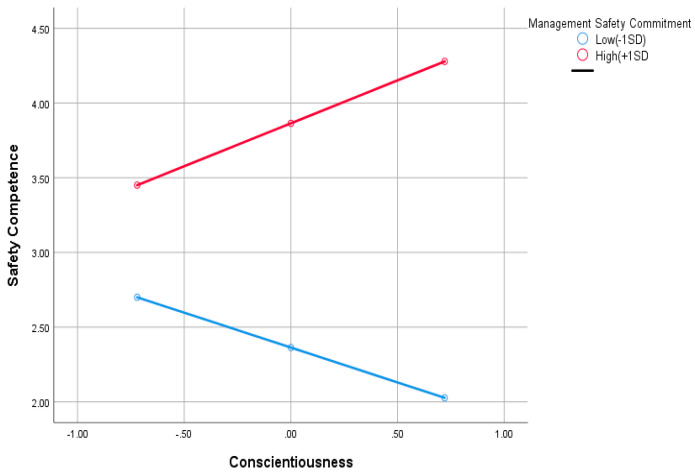
The moderation effect of management safety commitment on the relationship between conscientiousness and safety competency.

Hypothesis 3b tests the moderating influence of management safety commitment on the link between conscientiousness and safety competency. The interaction term for conscientiousness with the management safety commitment (β = 0.211, SE = 0.023, t = 9.1881, *p* = 0.00) is statistically significant in predicting safety competency. The significant interaction term provided evidence of the moderating effect of management safety commitment on the relationship between conscientiousness and safety competency. The moderating relationship is graphically shown in Figure 2. According to Figure 2, the link between conscientiousness and safety competency was stronger under high management safety commitment than lower levels of management safety commitment. The intensity of management’s commitment to safety improves in tandem with employees’ increasing conscientiousness so that higher levels of management’s safety commitment are linked to greater safety competency than those found at lower levels of management’s safety commitment. These findings confirm that Hypothesis 3b is supported.

Management safety commitment did not moderate the relationship between extraversion and safety competency; therefore, H3c is not supported.

**Figure 3:**
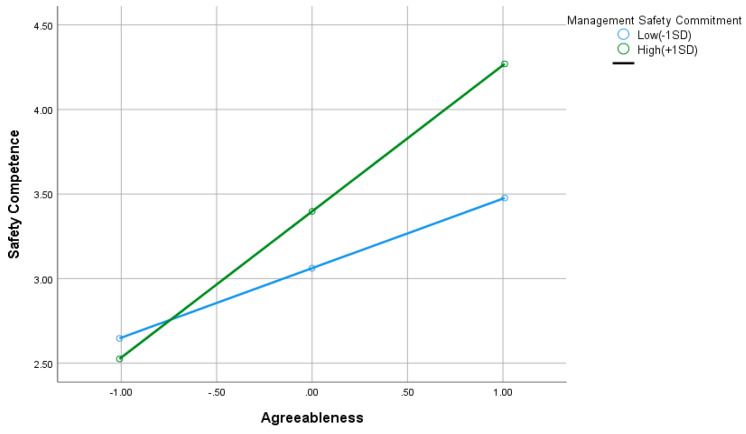
The moderation effect of management safety commitment on the relationship between agreeableness and safety competency.

Hypothesis 3d supposes that management safety commitment moderates the relationship between agreeableness and safety competency. In our analysis, management safety commitment was found to moderate the effect of agreeableness on safety competency (interaction β = 0.367, SE = 0.106, t = 3.267, *p* = 0.00, 95% CI = [0.138;0.555]), supporting H3d. As shown in Figure 3, high management commitment to safety is demonstrated by a steeper slope on the graph. At high management safety commitment, the effect of agreeableness on safety competency is significantly bigger. The line, however, tends to narrow at low management safety commitment, demonstrating that increasing degrees of agreeableness do not cause a corresponding shift in safety competency.

Moderation hypothesis 3e is not supported because management safety commitment does not moderate the relationship between neuroticism and safety competency. The interaction term was insignificant.

Additionally, we tested hypothesis 4 by looking at the conditional indirect effect (moderated mediation) of safety competency on the link between the personality traits variables and safety behavior at various degrees of management safety commitment. The conditional indirect effects estimates, standard errors, and bias-corrected percentile bootstrap confidence ranges are shown in Table 6.

The H4a hypothesis states that management safety commitment will moderate the indirect effect of openness on safety behavior through safety competency. Given that the 95% confidence interval (CI) includes zero, the index of moderated mediation (index = 0.013, 95% CI = [−0.103;0.128]) does not provide sufficient evidence to support H4a.

Findings also show that management safety commitment moderates the indirect impact of safety conscientiousness on safety behavior via safety competency. Management safety commitment is hypothesized to moderate the indirect effect of conscientiousness on safety behavior via safety competency (H4b). Because the index of moderated mediation (index = −0.168, 95% CI = [0.122;0.222]) is statistically significant, H4b was supported. The indirect effect was positive and significant for companies with high managerial safety commitment (mean + 1SD) (effect = 0.121, SE =0.045, 95% CI = [0.122;0.221]) and negative and statistically significant for companies with low management safety commitment (mean − 1SD) (effect = −0.098, SE = 0.028 95% CI = [−0.152;−0.044]). Thus, the conditional indirect effect of conscientiousness on safety behavior via safety competency increases with increasing levels of management commitment to safety. The effect increases from negative at low levels of management safety commitment to positive at a high level of management safety commitment.

Hypothesis H4c suggested that the indirect effect of extraversion on safety behavior through safety competency will be moderated by management Safety commitment. H4c was not supported as the index of moderated mediation (index = 0.018, 95% CI = [−0.019;0.064]) is insignificant since the confidence interval includes zero.

The moderated mediation hypothesis 4d model was supported with a significant index of moderated mediation (index = 0.075, 95% CI = [0.012;0.120]). Zero is not within the confidence interval; this signifies a significant moderating effect of management safety commitment on the indirect effect of safety competency. The conditional indirect effect was strongest in companies with high management safety commitment (mean + 1SD; effect = 0.190, SE = 0.025, 95% CI = [0.136;0.234]) and weak in companies low in management safety commitment (mean − 1SD, effect = 0.089, SE = 0.036, 95% CI = [0.027;0.173]). H4e was not supported because the index of moderated mediation was insignificant. Management safety commitment does not moderate the indirect effect of neuroticism on safety behavior through safety competency.

## 5. Discussion

Researchers have used both moderation and mediation to disclose the complexity of the interrelationships between a collection of variables and inquire into possible antecedents and outcomes. Moderated mediation has recently grown in popularity as a way to evaluate presumptions about the reasons or processes behind which a variable is linked to the desired result. We examined and demonstrated how moderated mediation analysis may be used to determine if an indirect impact depends on the values of a suggested moderating variable in this work. The study provides theoretical and empirical explanations for how personality traits (Openness, Conscientiousness, Extraversion, Agreeableness, and Neuroticism) affect safety behavior by establishing safety competency as a mediating variable and management safety commitment as a moderator. The data for the study was collected from 344 workers at six large-scale gold mining companies in Ghana.

We found that openness and safety behavior had a negative insignificant direct relationship. People who score highly on this feature might be predicted to have a great curiosity about different approaches and a correspondingly high propensity for unconventional actions and behavior [29]. Relatively open workers may therefore be cynical of the safety protocols made by the company and might not willingly obey them [41]. We initially hypothesized a negative relationship between openness and safety behavior. However, this hypothesis was not supported because the link was statistically insignificant. We did not find mediation or moderation effects on this variable.

The study also found that conscientiousness is positively related to safety behavior. The only personality trait that is obviously linked to adherence is conscientiousness because this trait is distinguished by self-control, which typically promotes conformity with standards and regulations. According to Wallace and Chen [44], persons with greater levels of conscientiousness frequently engage in more preventive or advantageous activities. At the same time, they are less likely to engage in unsafe behaviors at work [41]. Our finding is in line with Beus et al. [28], who found a strong connection between conscientiousness and safety behavior in different work contexts. We also found that safety competency partially mediates the relationship between conscientiousness and safety behavior.

Further analysis showed that employees’ perception of management safety commitment moderates the relationship between conscientiousness and safety competency. The indirect effect of safety competency on the relationship between conscientiousness and safety behavior is also moderated by safety competency. This brings to light that highly conscientious individuals are more likely to have a high level of safety competency in a work environment when the management makes safety a high priority. The indirect effect of safety competency is such that it increases from negative at low levels of management safety commitment to positive at high levels of management safety commitment. This further demonstrates that when leadership prioritizes safety, employees are more diligent and skilled in safety-related areas.

Extraversion was found to have an insignificant direct relation with safety behavior. On the other hand, Clarke and Roberson [70] found that extraversion negatively affects safety behavior. Mediation analysis showed that the relationship between extraversion and safety behavior is fully mediated by safety competency. Management safety commitment did not have a moderating effect on the relationship between extraversion and safety behavior. This finding demonstrates that in the Ghanaian mining sector, the safety competency of extrovert workers needs to be prioritized for their safety in the workplace. According to Tao et al. [37], extraverts are more susceptible to acting impulsively due to their highly variable behavioral patterns and the natural tendency for trying new things. Tao et al.’s assertion validates our finding that extraverts require a higher safety competency to ensure a safe work environment.

We have mentioned that agreeable people are more likely to comply with social norms and follow established procedures to avoid offending others. According to studies [39,41,43] examining the connections between personality qualities and safety behavior, agreeableness and safety behavior are positively correlated. These findings align with ours; we found a positive relationship between agreeableness and safety behavior partially mediated by safety competency. Moderation analysis revealed that management safety commitment regulates the relationship between agreeableness and safety competency. At high management safety commitment, the effect of agreeableness on safety competency is significantly bigger. However, at low management safety commitment, increasing levels of agreeableness do not cause a corresponding change in safety competency. Low management safety commitment does not affect the safety competency of agreeable individuals and does not affect their safety behavior.

Lastly, we found that neuroticism had a negative relation with safety behavior. According to the negative association between neuroticism and safety behavior, those with higher levels of neuroticism would act more recklessly and, consequently, less in accordance with workplace safety regulations [14]. This result is similar to that of Clarke and Roberson [27], who found a positive link between neuroticism and accident occurrence in different work contexts. It was also discovered that safety competency is an insignificant mediator in the relationship between neuroticism and safety behavior. Moderation results also showed that management safety commitment had moderation effects on the link between neuroticism and safety behavior.

These results demonstrate that highly conscientious and agreeable employees are likely to have high safety competencies, particularly in a company where management commitment to safety is equally high.

## 6. Limitations and Future Directions

Despite these limitations, the current study paves the way for more investigation in this area. First, we relied on self-reported measures of personality, which may not provide a reliable reflection of the true personality of the individual being evaluated. Our study also used participants’ perceptions of their safety competency and the management’s commitment to safety to measure these constructs, a methodology also followed by earlier studies. Although precautions were taken to eliminate the possibility of common method bias in this study, the self-evaluation could be affected by biases, underestimating, or overestimating. The conclusions should be explained cautiously.

Second, the data was collected from a survey of only six large-scale gold mining companies in Ghana using a convenience sampling approach, and the questionnaire was developed with literature from other fields. Therefore, there are limitations to taking these findings as indicative of the mining population at large. Third, while simultaneously testing the moderated mediating effects, the current study looked at safety competency as a mediator and management safety commitment as a moderator in the link between personality and safety behavior. Other mediating or moderating variables such as motivation, supervisor support, and certain organizational factors could be included by future researchers to advance their work.

Lastly, because we only looked at data from Ghana, our findings have little generalizability, especially concerning Western countries. Culture may influence personality, and the relationships between personality and safety practices may change among nations. According to Heine [71], culture and personality are inextricably linked. In other words, a person’s personality cannot be properly understood without taking their cultural environment into account. People’s safety behavior and attitudes to risk vary according to their values and beliefs [72]. Based on the importance attached to the need to maximize safety, developed western countries are seen to be stricter about workplace safety [73]. In such an environment, workers will engage in safe work behaviors. Moreover, the mining environment in Ghana is more complicated than in Western countries because of people’s poor compliance with safety requirements, lack of safety knowledge, and weak safety legislation [73]. Due to variations in culture, society, and the workplace, evidence-based recommendations from Ghana may not be appropriate for western nations. We therefore suggest that researchers conduct similar research in various cultural settings.

## 7. Conclusions

We conducted this study to learn more about the relationship between personality traits and safety behavior. We provided proof of the effects of personality on safety behaviors among gold mine employees in Ghana. We demonstrated that this connection is more sophisticated than generally anticipated as safety competency was a significant mediator in this interaction. Therefore, maintaining a high level of safety competency is essential for determining whether or not workers follow safety procedures, which impacts the effectiveness of creating a safe working environment. The study has made us aware of the difficulty companies face when creating and enforcing safety regulations, given the different responses they may receive from employees with different personalities. Since various personality traits impact one’s degree of safety competency, managers should be aware that some workers may not find the rules and techniques for enforcing safety standards to their liking. Various tactics may need to be implemented to promote compliance with safety measures.

Companies that want to improve safety behaviors could provide safety training programs to promote safety proficiency and awareness campaigns on the significance of safety [74]. Second, companies should consider agreeableness, extraversion, and conscientiousness when developing and implementing operational safety procedures, considering the relationship of these traits with safety behavior. Considering personality types may help select workers more likely to act responsibly and value workplace safety. It would be beneficial to allocate work to people based on their higher-order personalities; those who are very conscientious or pleasant may be more likely to follow safety rules and be given more complex assignments. Neurotic employees who are more inclined to participate in risky behavior can be assigned tasks with minimal risk.

The study also revealed the importance of management that has a high commitment to the safety of employees. Management must be ready to make safe investments to guarantee fewer accidents and fatalities and demonstrate genuine concern for their workers [53]. A company needs formal regulatory requirements to ensure safe production, especially when there is no standard process for how a certain activity is to be conducted or if the usual procedures are inadequate for the safe execution of the job. Companies should have a documented and dated hazard assessment prepared by management once an inspection has uncovered any potential or actual dangers [10]. When there are modifications to the job’s requirements, tools, or surroundings, it is essential to revisit the hazard assessment. Standardizing treatment procedures, developing procedure manuals, and removing unnecessary variations are all ways to lower accident and injury rates [10]. These processes ensure that tasks are executed safely and effectively and may lead to an improved and more secure workplace.

Furthermore, management could develop safety training programs to address traits like openness and neuroticism that could increase risk-taking and unsafe behavior. They should make it a priority to root out negative employees in the course of their regular monitoring, consulting, and communication. For instance, businesses may provide coaches or therapists to workers who exhibit early indicators of emotional instability to assist them to stop thinking incessantly and negatively. Ignoring these crucial personality traits threatens a company’s output efficiency and general safety.

The framework and survey employed in this study are well-structured and have a high level of reliability. The findings can be used as a foundation for future investigations into the safety behavior of gold miners and as a point of reference for empirical analyses. The personality/safety behavior model we created has significant ramifications for corporate safety managers. Businesses must first improve staff safety competency to raise the likelihood of safe activities. This can be accomplished through instruction, efficient incentive and punishment schemes, or publicity highlighting the need for safety. It is crucial to frequently train staff members on workplace safety procedures. Workers gain from instruction that clarifies safety procedures, risk management techniques, response strategies, and other crucial subjects.

## Figures and Tables

**Table 1:** Demographic Details of Respondents.

		Frequency	Percentage
Gender	Male	263	76.5%
	Female	81	23.5%
Age	18–29	110	32.0%
	30–40	150	43.6%
	41–50	60	17.4%
	51–60	24	7.0%
Marital Status	Single	83	24.1%
	Married	213	61.9%
	Separated	28	8.1%
	Divorced	11	3.2%
	Widowed	9	2.6%
Education	Senior high or below	32	9.3%
	Bachelor’s	192	55.8%
	Master’s	101	29.4%
	Doctoral or higher	19	5.5%
Work Experience	Less than two years	45	13.1%
	2 to 5 years	139	40.4%
	6 to 10 years	108	31.4%
	More than 10 years	52	15.1%

**Table 2:** Standard Deviations, Means, and Correlations.

	Gen	Age	Mar	Edu	Work	OP	CON	EX	AG	NR	SB	SC	MSC
Gen	1												
Age	−0.027	1											
Mar	−0.013	0.255 **	1										
Edu	0.136 *	0.313 **	0.073	1									
Work	−0.010	0.333 **	0.140	0.145 **	1								
OP	−0.032	0.071	0.008	0.032	0.051	1							
CON	−0.010	−0.008	−0.050	0.074	−0.004	0.047	1						
EX	−0.060	0.041	−0.029	0.014	0.045	0.064	0.203 **	1					
AG	−0.052	−0.002	−0.053	−0.046	0.006	0.119 *	0.584 **	0.597 **	1				
NR	0.044	−0.052	−0.020	−0.026	−0.008	0.012	0.013	0.011	0.064	1			
SB	−0.025	0.018	−0.047	0.042	0.005	0.026	0.642 **	0.172 **	0.483 **	0.805 **	1		
SC	−0.049	−0.003	−0.031	0.087	0.021	0.049	0.316 **	0.411 **	0.670 **	0.610 **	0.580 **	1	
MSC	−0.028	0.042	0.019	0.035	−0.010	0.077	0.725 **	0.546 **	0.683 **	0.633 **	0.722 **	0.539 **	1
Mean	1.24	1.99	1.98	2.31	2.49	2.825	4.132	3.442	3.370	3.463	4.353	3.385	3.936
Std Dev.	0.425	0.881	0.829	0.716	0.903	1.088	0.721	0.951	1.009	0.665	0.633	1.347	0.653

**, * signifies that correlation is significant at the 0.01 and 0.05 levels, respectively. Gen = gender, Mar = marital status Edu = Education, Work = work experience, OP = openness, CON = conscientiousness, EX = extraversion, AG = agreeableness, NR = neuroticism, SB = Safety behavior, SC = safety competency, and MSC = management safety commitment.

**Table 3:** Recommended Values for Model Fit Indexes.

Model Fit Index	Recommended Value
χ2/df	1–3 [66]
GFI	≥0.9 [67,68]
CFI	≥0.95 [61,69]
TLI	≥0.95 [61,69]
RMSEA	<0.06 acceptable, ≥0.10 bad [61,69]
SRMR	<0.08 [61]

**Table 4:** Results of Confirmatory Factor Analysis.

Model	χ^2^/df	CFI	GFI	TLI	RMSEA	SRMR
Eight-factor model (OP, CON, EX, AG, NR, SB, SC, MSC)	1.395	0.963	0.917	0.956	0.032	0.015
Five-factor model (OP + CON + EX + AG + NR, SP, SCO, SC, MSC)	2.453	0.891	0.873	0.851	0.095	0.073
Four-factor model (OP + CON + EX + AG + NR, SB, SC, MSC)	3.538	0.846	0.832	0.80	0.13	0.085

OP = openness, CON = conscientiousness, EX = extraversion, AG = agreeableness, NR = neuroticism, SP = Safety participation, SCO = safety compliance, SC = safety competency, and MSC = management safety commitment.

**Table 5:** Results of Mediation Analysis.

		Effect	SE	t	p	LLCI	ULCI
Total Effects	OP > SB	0.015	0.032	0.483	0.630	−0.047	0.077
	CON > SB	0.564	0.036	15.485	0.000	0.493	0.636
	EX > SB	0.115	0.036	3.237	0.001	0.045	0.185
	AG > SB	0.303	0.030	10.209	0.000	0.245	0.362
	NR > SB	−0.273	0.021	13.158	0.000	−0.232	−0.313
Direct Effects		**Effect**	**SE**	**t**	**p**	**LLCI**	**ULCI**
	OP > SB	−0.001	0.026	−0.049	0.961	−0.052	0.049
	CON > SB	0.449	0.033	18.622	0.000	0.383	0.513
	EX > SB	0.053	0.032	−1.648	0.100	−0.116	0.010
	AG > SB	0.108	0.037	2.932	0.004	0.036	0.181
	NR > SB	−0.271	0.021	13.061	0.000	−0.230	−0.312
Indirect Effects		**Effect**	**BootSE**	**BootLLCI**	**BootULCI**	**Result**	
	OP > SC > SB	0.017	0.018	−0.019	0.052	No Mediation	
	CON > SC < SB	0.116	0.029	0.070	0.182	Partial Mediation	
	EX > SC < SB	0.168	0.019	0.132	0.205	Full Mediation	
	AG > SC > SB	0.195	0.035	0.130	0.268	Partial Mediation	
	NR > SC > SB	−0.002	0.002	−0.002	0.006	No Mediation	

SE = standard error, LLCI = low level of confidence interval, ULCI = upper level of confidence interval, OP = openness, CON = conscientiousness, EX = extraversion, AG = agreeableness, NR = neuroticism, SB = Safety behavior, SC = safety competency.

**Table 6:** Results of Moderated Mediation Analysis.

Variable	Mediator	Effect	BootSE	BootLLCI	BootULCI	Result
OP	Low level of MSC	−0.001	0.052	−0.102	0.104	
	High level of MSC	0.016	0.037	−0.059	0.088	Insignificant
	Index of Moderated Mediation	0.013	0.059	−0.103	0.128	Moderated Mediation
CON	Low level of MSC	−0.098	0.028	−0.152	−0.044	
	High level of MSC	0.121	0.045	0.122	0.221	Significant
	Index of Moderated Mediation	0.168	0.025	0.122	0.222	Moderated Mediation
EX	Low level of MSC	0.021	0.019	−0.021	0.053	
	High level of MSC	0.045	0.017	0.013	0.081	Insignificant
	Index of Moderated Mediation	0.018	0.021	−0.019	0.064	Moderated Mediation
AG	Low level of MSC	0.089	0.036	0.027	0.173	
	High level of MSC	0.190	0.025	0.136	0.234	Significant
	Index of Moderated Mediation	0.075	0.025	0.021	0.120	Moderated Mediation
NR	Low level of MSC	0.003	0.028	−0.051	0.060	
	High level of MSC	0.018	0.022	−0.028	0.060	Insignificant
	Index of Moderated Mediation	0.111	0.032	−0.054	0.072	Moderated Mediation

SE = standard error, LLCI = low level of confidence interval, ULCI = upper level of confidence interval, OP = openness, CON = conscientiousness, EX = extraversion, AG = agreeableness, NR = neuroticism, MSC = management safety commitment.

## Data Availability

The data presented in this study are available upon request from the corresponding author.
